# Lactate Suppresses Growth of Esophageal Adenocarcinoma Patient-Derived Organoids through Alterations in Tumor NADH/NAD+ Redox State

**DOI:** 10.3390/biom14091195

**Published:** 2024-09-22

**Authors:** Steven H. Su, Yosuke Mitani, Tianxia Li, Uma Sachdeva, Samuel Flashner, Andres Klein-Szanto, Karen J. Dunbar, Julian Abrams, Hiroshi Nakagawa, Joel Gabre

**Affiliations:** 1 Department of Medicine, Columbia University Irving Medical Center, New York, NY 10032, USA; 2 Columbia University Herbert Irving Comprehensive Cancer Center, New York, NY 10032, USA; 3 Division of Digestive and Liver Diseases, Department of Medicine, Columbia University Irving Medical Center, New York, NY 10032, USA; 4 Columbia University Digestive and Liver Diseases Research Center, Columbia University Irving Medical Center, New York, NY 10032, USA; 5 Division of Thoracic Surgery, Massachusetts General Hospital, Boston, MA 02114, USA; 6 Histopathology Facility, Fox Chase Cancer Center, Philadelphia, PA 19111, USA

**Keywords:** lactate, esophageal adenocarcinoma, tumor microenvironment

## Abstract

Barrett’s esophagus (BE) is a common precancerous lesion that can progress to esophageal adenocarcinoma (EAC). There are significant alterations in the esophageal microbiome in the progression from healthy esophagus to BE to EAC, including an increased abundance of a variety of lactate-producing bacteria and an increase of lactate in the tumor microenvironment, as predicted by metabolic modeling. The role of bacterial lactate in EAC is unknown. Here, we utilize patient-derived organoid (PDO) models of EAC and demonstrate that lactate inhibits the growth and proliferation of EAC PDOs through alterations in the tumor NADH/NAD+ redox state. Further RNA sequencing of EAC PDOs identifies ID1 and RSAD2 as potential regulatory molecules crucial in mediating lactate’s ability to suppress glycolysis and proliferation. Gene ontology analysis also identifies the activation of inflammatory and immunological pathways in addition to alterations in the metabolic pathways in EAC PDOs exposed to lactate, suggesting a multi-faceted role for lactate in the pathogenesis of EAC.

## 1. Introduction

Barrett’s esophagus (BE) results from repeated acid-induced injury to esophageal epithelium at the gastro-esophageal junction, commonly due to gastroesophageal reflux disease (GERD) and can progress to esophageal adenocarcinoma (EAC). Both BE and EAC are rising in incidence worldwide, with the incidence of BE increasing five to seven times now compared to 30 years prior [1,2,3]. It is estimated that about 2% of all adults worldwide suffer from BE with an additional 0.5–1% of those going on to develop EAC [4]. Pathologically, BE is characterized by metaplastic changes in the normal esophageal stratified squamous epithelium to columnar epithelium, with increasing preneoplastic morphological changes (i.e., dysplasia) leading to EAC [4].

The gut microbiome may have a critical role during carcinogenesis by modulating host antitumor immunity, inflammation, energetics, and cell signaling [5]. Interestingly, our group and others have observed significant alterations in the esophageal microbiome in BE and EAC compared to healthy human esophagus [6,7]. In particular, there is a dramatic increase in multiple lactate-producing *Streptococcus*, *Lactobacillus*, and *Bifidobacterium* species [7,8,9,10,11], as corroborated by metabolic modeling, which predicts an increase in bacterial lactate levels from healthy esophagus to BE to EAC [8].

The effect of bacterial-derived metabolites, including lactate, has not been well characterized in BE and EAC. L-lactate, one of the two isomers (enantiomers) of lactate [12], is the predominant isomer produced by *Streptococcus* and *Bifidobacterium* species and is also produced by *Lactobacillus* species, all of which have increased abundance in the EAC microbiome compared to healthy esophagus [13]. Notably, L-lactate is also produced by healthy mammalian cells during anaerobic glycolysis. In cancerous cells, glycolytic machinery can be reprogrammed such that there is increased glucose uptake to facilitate cellular lactate production. This is known as “aerobic glycolysis” or the Warburg effect and has been seen in breast, colon, cervical, and liver cancers [12]. Specifically, the Warburg effect is a phenomenon whereby cancer cells rely primarily on cytosolic glycolysis and lactate fermentation rather than oxidative phosphorylation, even in the presence of oxygen [12]. Given the predicted increase in bacterial L-lactate in the tissue microenvironment during the dysplastic progression of the healthy esophagus to BE and from BE to EAC [8], we explored the role of L-lactate in these pathogenic changes utilizing patient-derived organoid (PDO) models of EAC that recapitulate morphology, molecular attributes, and functions of original tissues [14]. Herein, we demonstrate that L-lactate alters tumor NADH/NAD+ redox state to regulate EAC growth and proliferation.

## 2. Materials and Methods

### 2.1. Tissue Procurement

Tissue procurement and generation of PDO lines were previously approved by the University of Pennsylvania Institutional Review Board (IRB protocol #813841) and by the Columbia University Institutional Review Board (IRB #AAAS4603). Informed consent was obtained for biopsy specimens from patients undergoing diagnostic endoscopy for suspected esophageal cancer. All methods were performed in accordance with Columbia University and the University of Pennsylvania IRB committees’ regulations on human subject research.

### 2.2. Generation and Passaging of Esophageal Adenocarcinoma Patient-Derived Organoids

EAC PDOs were generated from endoscopic biopsies procured at the Columbia University Irving Medical Center under the approved IRB protocol (AAAT8778) and the University of Pennsylvania under IRB approval (#813841) and passaged as previously described [14]. Briefly, tissues were washed with PBS, digested with Dispase (Corning, Corning, NY, USA 354235) and 0.25% Trypsin-EDTA (Gibco, Waltham, MA, USA 25200056), and then the cell suspension was passaged through a 100 µm cell strainer (Falcon, Corning, NY, USA). Trypsin was then inactivated with a soybean trypsin inhibitor (Sigma-Aldrich, St. Louis, MO, USA T9128). After centrifuging the cells and aspirating the supernatant, the cell pellet was washed in cold PBS and resuspended in cold PBS. Cells were counted and then seeded at between 2500 and 5000 cells per 50 µL of Matrigel (Corning, Corning, NY, USA 354234), each in a 24-well plate. The plate was placed at 37 °C for 20 min to allow solidification of the Matrigel and then covered in media. To passage PDOs, Matrigel domes with PDOs were mechanically dissociated via pipetting with cold PBS. The mixture was then treated with 0.05% Trypsin and incubated. Trypsin was inactivated with the addition of soybean trypsin inhibitor, and then the cells were strained through a 75 µm cell strainer. The mixture was then centrifuged and then the supernatant was aspirated. The cell pellet was resuspended in cold PBS, cells were counted, and 2000–5000 single cells were seeded in a Matrigel dome in a 24-well plate. After the solidification of the Matrigel, media was added.

### 2.3. Clinical/Pathologic Features of Tumor Samples

See Supplemental Figure S6 for details regarding the clinical and pathologic features of the tumor/PDO samples. EAC000 and EAC011 have been previously characterized [14].

### 2.4. Formulation of Normal Glucose and Low-Glucose Human Gut Media

Normal glucose human gut media was prepared as previously described [14]. Briefly, normal glucose media contained 50% by volume Advanced D-MEM/F-12 (Gibco, Waltham, MA, USA 12634010), 50% by volume WRN conditioned media [14], 1x Glutamax (Gibco, Waltham, MA, USA 35050061), 10 mM HEPES (Gibco, Waltham, MA, USA 15630130), 1x N-2 supplement (Gibco, Waltham, MA, USA 17502048), 1x B-27 supplement (Gibco, 17504044), 1 mM N-Acetylcysteine (Sigma-Aldrich, St. Louis, MO, USA A9165), 0.5 µM CHIR99021 (Cayman Chemicals, Ann Arbor, MI, USA 13122), 250 ng/mL Human EGF (PeproTech, Waltham, MA, USA 100–15), 0.5 µM A83-01 (Cayman Chemicals, Ann Arbor, MI, USA 9001799), 1 µM SB202190 (Selleck Chemicals), 0.1 µM Gastrin (Sigma-Aldrich A9165), 4 mM Nicatinamide (Sigma-Aldrich, N0636), 10 µM Y-27643 (Selleck Chemicals, Houston, TX, USA S1049), and 1x Antibiotic-Antimycotic (Gibco, Waltham, MA, USA 15240062). A total of 100 ng/mL FGF-10 (PeproTech, 100-26) was added only when generating PDOs from primary cultures. The formulation for low-glucose human gut media was identical to that of normal glucose human gut media except 50% SILAC Advanced D-MEM/F-12 Flex Media No Glucose No Phenol Red (Gibco, A24494301) was substituted for Advanced D-MEM/F-12. Additionally, 147.5 mg/L L-Arginine Hydrochloride (Thermo Scientific, Waltham, MA, USA 105000250) and 91.25 mg/L L-Lysine Monohydrochloride (Sigma-Aldrich, St. Louis, MO, USA L5626-100g) were supplemented to the low-glucose human gut media given the absence of the amino acids in the SILAC Advanced D-MEM/F-12 Flex Media compared to regular Advanced D-MEM/F-12. Final glucose concentrations in the normal and low-glucose human gut media were 17.5 mM and 8.75 mM.

### 2.5. Lactate Treatment of PDOs

In total, 20 mM Sodium L-lactate (Sigma-Aldrich, St. Louis, MO, USA L7022) was added to the media conditions described above. PDOs were grown for the specified durations as described above in each individual experiment (typically 14 days). Lactate-containing media was exchanged every 2–3 days.

### 2.6. Quantification of PDO Size and Organoid Formation Rate

PDOs were examined under Celigo Image Cytometer (Nexcelom, Lawrence, MA, USA) at desired time points. The average PDO area was calculated by Celigo per well, and these average areas were then pooled together and used for statistical testing. The Celigo Image Cytometer was also used to quantify the number of PDOs per well, and the organoid formation rate of each well was computed as OFR = number of PDOs/number of seeded cells. The OFRs of each of the wells were then pooled together and used for statistical testing.

### 2.7. Extraction of PDOs for Immunohistochemistry, Immunofluorescence, and RNAseq

PDOs were extracted from Matrigel as previously described [14]. Briefly, media was aspirated from each well and replaced with 500 µL of PBS. The Matrigel was mechanically dissociated via gentle pipetting. Appropriate wells were combined together to expand the quantity of PDOs. The PDOs were then centrifuged on a bench-top mini-centrifuge for 30 s. The supernatant was aspirated and either resuspended in PBS (for RNAseq) or resuspended in 4% PFA and fixed overnight (for Immunohistochemistry and Immunofluorescence).

### 2.8. Paraffin Embedding of PDOs for Immunohistochemistry and Immunofluorescence

Paraffin embedding was performed as previously described [14]. Briefly following fixation, PDOs were centrifuged, and the PFA solution was aspirated. In total, 50 µL embedding gel was added to each PDO pellet, and then the PDOs were resuspended in the gel. The solution was then placed on an embedding rack and cooled at 4C to solidify the gel into a bubble. After solidification, the PDO gel bubbles were placed in cassettes and stored in 70% ethanol until embedded in paraffin. Paraffin embedding of the PDO gel bubbles was performed on a Leica EG1150 Tissue Embedding Center (Leica, Nussloch, Germany). The paraffin blocks were then sectioned into 10 µm sections onto standard microscopy slides using a manual rotary microtome (Leica, Nussloch, Germany).

### 2.9. H&E, Immunofluorescence and Alcian Blue Staining of PDOs

IHC and IF of paraffin slides were performed as previously described [14]. Briefly paraffinized sections on slides were de-paraffinized by warming on a slide warmer, immersing in a Xylene bath, and subsequently submersing in graded ethanol baths (100%, 95% ×2, 80%, 70%, deionized water). Antigen retrieval was performed by heating slides in 10 mM Citric Acid (pH 6.0) for 15 min. Slides were washed in water and then PBS. H&E staining was completed as previously described [14]. For immunofluorescent staining, regions of interest were restricted by marking with a hydrophobic pen. Slides were blocked in a Starting Block T20 Blocking Buffer (Thermo Scientific, Waltham, MA, USA 37539). After blocking, primary antibodies were diluted in Starting Block T20 Blocking Buffer at dilutions listed below, applied to slides, and then incubated overnight at 4C. The next morning, the primary antibody was washed off, and secondary antibodies diluted in Starting Block T20 blocking buffer were applied to slides and incubated at room temperature (covered) for 2–3 h. The secondary antibody was then washed off with PBS and slides were mounted with ProLong Gold Antifade Mountant with DAPI (Invitrogen, Waltham, MA, USA 36935) with a coverslip. Alcian Blue staining was completed using Alcian Blue (pH 2.5) Stain Kit (Vector Laboratories, Newark, CA, USA H-3501) per manufacturer instructions.

### 2.10. Antibodies and Dilutions

Rabbit anti-Ki67 (Abcam, Cambridge, UK 16667, 1:200), Rabbit anti-cleaved-Caspase3 (ASP 175) (5A1E) (Cell Signaling Technology, Danvers, MA, USA 9661S, 1:200), Rabbit anti-Cytokeratin 8 (Abcam, Cambridge, UK 59400, 1:200), and Alcian Blue (Vector Labs, Newark, CA, USA H-3501).

### 2.11. Immunofluorescent Imaging of PDOs

Immunofluorescent images of PDOs were captured using a Keyence BZ-X All-in-one Fluorescence Microscope (Keyence, Itasca, IL, USA).

### 2.12. Quantification of Ki67, CDX2 and Cytokeratin 8 Immunofluorescence

For each condition, >3 Images of PDOs were captured at 20× such that there were multiple PDOs per image and between approximately 200–1000 cells per image. Using ImageJ version 1.53 (https://imagej.net), the total number of cells per image was counted via DAPI staining, and the number of cells positive for Ki67, CDX2, or Cytokeratin 8 was also counted. For each image, a percentage of positive cells was computed. These percentages were then pooled across images and used for statistical testing.

### 2.13. Histological Scoring of Esophageal Adenocarcinoma PDOs

Scoring was performed using the following metrics. Score 1: no or mild nuclear atypia, numerous vacuoles, or glandular structure per organoid (approximately >6). Score 2: moderate nuclear atypia, numerous vacuoles, or glandular structure per organoid (approximately >6). Score 3: moderate to severe atypia, some vacuoles or gland structures per organoid (2–6). Score 4: severe nuclear atypia, very few or no vacuoles or gland structures per organoid (0–2)

### 2.14. NADH/NAD+ Quantification Assay

NADH and NAD+ were quantified using a NAD/NADH Quantitation Kit (Sigma Aldrich, St. Louis, MO, USA MAK037) per manufacturer instructions. The NADH/NAD+ ratio was calculated per manufacturer instructions. For statistical analysis, each condition was repeated 3 independent times and normalized to the control from the same experiment. The normalized values were then pooled for statistical analysis.

### 2.15. RNA Sequencing

Total RNA was extracted from organoids using the RNAqueous Micro Kit (Invitrogen, Waltham, MA, USA AM1931) and sequenced by Azenta (Burlington, MA, USA). In brief, RNA-seq libraries were prepared using the NEBNext Ultra RNA Library Prep Kit (New England Biolabs, Ipswich, MA, USA E7530) according to the manufacturer’s instructions, and sequencing was performed on an Illumina NovaSeq (San Diego, CA, USA), generating paired-end reads. The raw sequencing data were processed and analyzed using Azenta’s standard bioinformatics pipeline, which includes quality control, read alignment to the reference genome, transcript assembly, and differential expression analysis. Differentially expressed genes were identified using the DESeq2 package version 3.19. Further functional enrichment analysis was performed using Gene Ontology (GO) and Kyoto Encyclopedia of Genes and Genomes (KEGG) pathway analysis to interpret the biological significance of the differentially expressed genes.

## 3. Results

### 3.1. Lactate Suppresses EAC PDO Size but Not Formation Rate

**Figure 1:**
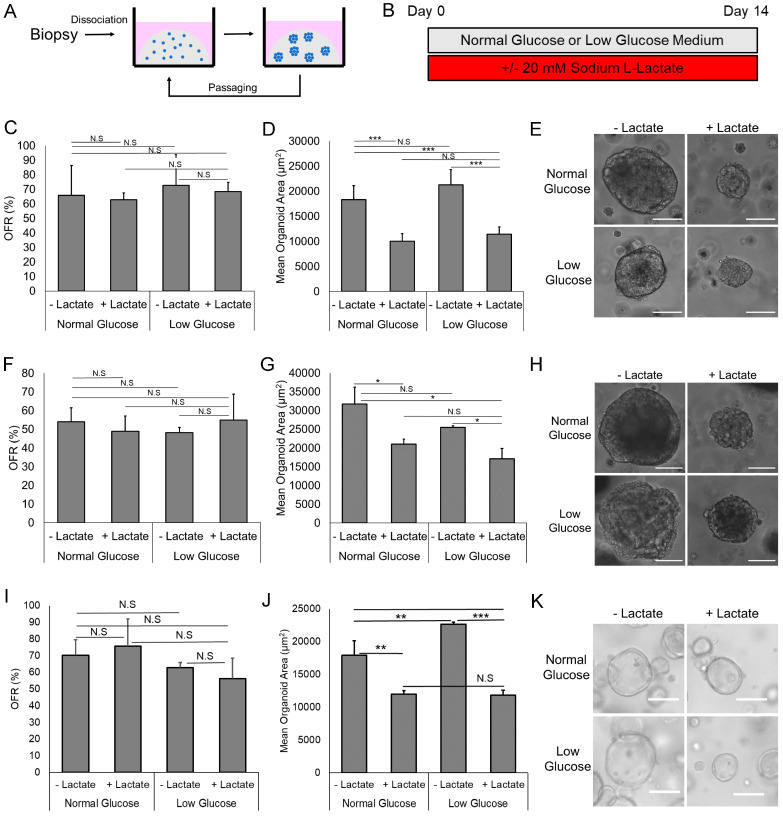
Lactate treatment decreases mean organoid area of EAC PDOs but has no effect on organoid formation rate (OFR). (**A**) Schematic of generation of patient-derived organoids. Biopsies were dissociated into single-cell suspensions and grown in Matrigel into esophageal organoids, after which they could be expanded and re-seeded as necessary. (**B**) Experimental schematic indicating EAC organoid growth conditions. (**C**) Organoid formation rate for EAC000 organoids grown in listed conditions. (**D**) Mean organoid area for EAC000 organoids grown in listed conditions. (**E**) Images of EAC000 organoids grown in listed conditions. (**F**) Organoid formation rate for HNEC001 organoids grown in listed conditions. (**G**) Mean organoid area for HNEC001 organoids grown in listed conditions. (**H**) Images of HNEC001 organoids grown in listed conditions. (**I**) Organoid formation rate for EAC011 organoids grown in listed conditions. (**J**) Mean organoid area for EAC011 organoids grown in listed conditions. (**K**) Images of EAC011 organoids grown in listed conditions. Error bars in (**C**,**D**,**F**,**G**,**I**,**J)** indicate standard deviations. Scale bars in (**E**,**H**,**K**) are 100 μm. *n* = 5 or 6 technical replicates for all measurements in (**C**,**D**,**F**,**G**,**I**,**J**). N.S indicates non-significant. * indicates *p* < 0.05 by Student’s *t*-test. ** indicates *p* < 0.01 by Student’s *t*-test. *** indicates *p* < 0.001 by Student’s *t*-test.

To determine the effect of lactate in EAC PDOs, PDOs were grown in the presence or absence of 20 mM Sodium L-lactate for 14 days, after which the size, organoid formation rate, morphologies, intestinal metaplastic markers, and gene expression were analyzed (Figure 1A,B). An amount of 20 mM lactate was chosen as a physiologically relevant concentration as prior studies indicate a concentration of 10–30 mM lactate in the tumor microenvironment [15].

We first utilized EAC000, a moderately differentiated EAC PDO line [14]. Interestingly, after 14 days of lactate exposure, we found that treatment of EAC000 PDOs with lactate resulted in significantly smaller organoids as quantified by mean organoid area, but that lactate had no effect on the organoid formation rate (OFR), a measure indicating the frequency of the cells that can give rise to organoids (e.g., tumor-initiating cells) (Figure 1C–E) [16,17,18]. This phenotype was observed when organoids were grown in 2 different glucose conditions: 17.5 mM glucose (“normal”)—the concentration at which PDOs have been previously cultured, as well as 8.25 mM glucose (“low”) (Figure 1C–E). This indicates that the observed size phenotype may be independent of environmental glucose concentrations. When we utilized HNEC001, another EAC PDO line displaying unique signet ring cell differentiation, we again found a similar phenotype whereby lactate treatment resulted in smaller PDOs but had no effect on the OFR (Figure 1F–H). This phenotype was also confirmed in a third EAC PDO line, EAC011, a poorly differentiated EAC (Figure 1I–K) [14]. When we examined earlier time points, this phenotype was already observable on day 10 of lactate treatment in EAC000 PDOs (Supplemental Figure S1), indicative that size phenotype is progressive during the growth of EAC PDOs.

Given our observation that lactate limits organoid size but has no effect on the OFR, considered a surrogate for tumor initiation from single cells [16,17,18], we next evaluated if the size phenotype is requisite on the presence of lactate at the initiation of PDO growth from single cells. When EAC000 PDOs were initially grown from single cells for 5 days in the absence of lactate and then subjected to lactate treatment after day 5, when PDOs had already formed from single cells, we observed that PDOs treated with lactate were still smaller in size compared to control (Supplemental Figure S2). Consistent with prior results, there was also no change in the OFR (Supplemental Figure S2). These data suggest that the observed size difference following lactate treatment occurs independent of the initiation of PDOs from single cells.

Notably, the addition of 20 mM lactate did not alter the pH of the growth medium (Supplemental Figure S3), suggesting that the size phenotype we observed is not due to alterations in environmental pH.

### 3.2. Lactate Reduces Proliferation but Does Not Increase Apoptosis

**Figure 2:**
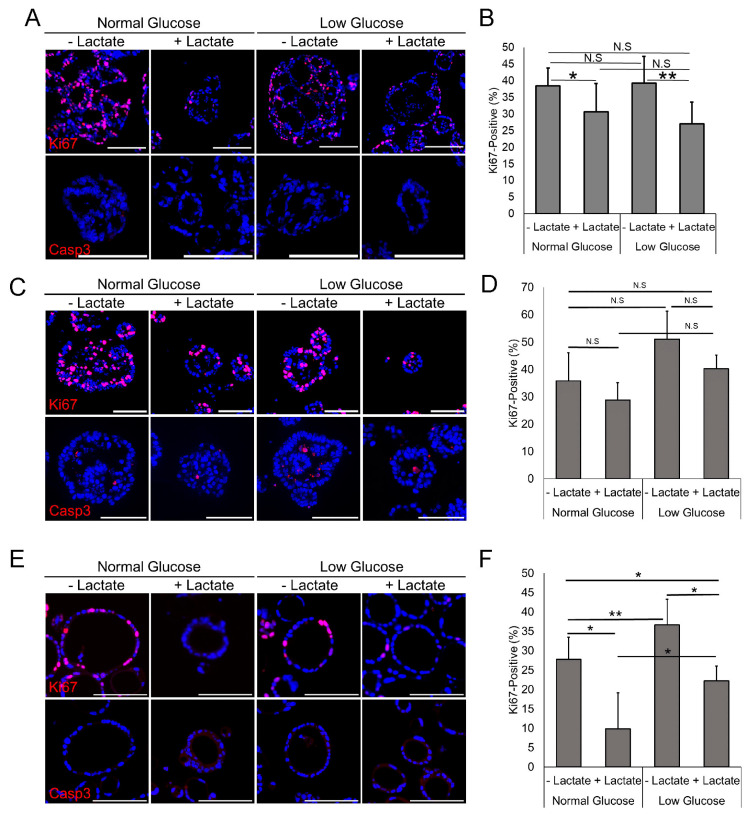
Lactate treatment decreases cellular proliferation in EAC PDOs. (**A**) Immunofluorescent staining of Ki67 and Cleaved Caspase 3 (Casp3) in EAC000 PDOs grown under various conditions. (**B**) Quantification of Ki67 positivity in EAC000 grown under various conditions. (**C**) Immunofluorescent staining of Ki67 and Cleaved Caspase 3 (Casp3) in HNEC001 PDOs grown under various conditions. (**D**) Quantification of Ki67 positivity in HNEC001 PDOs grown under various conditions. (**E**) Immunofluorescent staining of Ki67 and Cleaved Caspase 3 (Casp3) in EAC011 PDOs grown under various conditions (**F**) Quantification of Ki67 positivity in EAC011 PDOs grown under various conditions Scale bars in (**A**,**C**,**E**) are 100 μm. Error bars in (**B**,**D**,**F**) are standard deviations. * indicates *p* < 0.05 via Student’s *t*-test. ** indicates *p* < 0.01 via Student’s *t*-test. N.S indicates not significant. N = 3 to 8 medium-powered fields quantified for each condition in (**B**,**D**,**F**), with each field containing approximately 100–1000 nuclei.

Given our observation that lactate treatment results in smaller-sized EAC PDOs, we next wanted to evaluate if lactate could be affecting the proliferation or apoptosis of EAC PDOs. We performed immunofluorescence staining of EAC PDOs for cellular proliferation marker Ki67 and apoptosis marker Cleaved Caspase 3. In EAC000 PDOs, we found that lactate treatment resulted in a significant decrease in Ki67 expression but no change in Caspase3 expression (Figure 2A,B). This result is consistent across both glucose concentrations, indicating that the reduction in cellular proliferation is not likely due to insufficient environmental glucose. We found an identical result in EAC011 PDOs, whereby lactate-treated organoids also demonstrated significantly reduced cellular proliferation as indicated by Ki67 without a change in Caspase3 expression across all glucose concentrations (Figure 2E,F). A similar trend is also observed in HNEC001 PDOs following lactate treatment (Figure 2C,D). These data suggest that excess lactate suppresses cellular proliferation but does not induce apoptosis in EAC PDOs.

### 3.3. Lactate Has Variable Effect on the Cellular Atypia in EAC PDOs

Examination of histological sections of EAC PDOs demonstrated that lactate treatment has variable effects on the degree of cellular atypia in the PDOs. Specifically, lactate-treated and control EAC PDOs were scored 1 to 4, with a score of 1 indicating minimal nuclear atypia and well-differentiated EAC (numerous vacuoles/glandular structures) and a score of 4 indicating severe nuclear atypia and poorly differentiated EAC (see Materials and Methods for details). Using this scoring system, we found that lactate treatment does not affect the distribution of scores in EAC000 PDOs grown in either glucose condition. However, in HNEC001, lactate treatment resulted in high scores in both glucose conditions, indicating increased atypia and poorer differentiation status (Supplemental Figure S4). Interestingly, low glucose conditions in both EAC000 and HNEC001 resulted in significantly increased cellular atypia when compared to normal glucose controls (Supplemental Figure S4). Notably, when we performed immunofluorescent staining of various markers for intestinal metaplasia, including CDX2 and Cytokeratin 8, we found no difference in expression in either EAC000, HNEC001, or EAC011 PDOs after treatment with lactate compared to control (Supplemental Figure S5). When we stain for Alcian Blue (a marker of mucin-producing goblet/goblet-like cells), we also observe no difference in staining in either EAC000 or HNEC001 PDOs following treatment with lactate when compared to control (Supplemental Figure S5). Notably, Alcian Blue staining was absent in HNEC001 PDOs regardless of lactate treatment (Supplemental Figure S5). The negative staining in this signet ring cell line is likely due to the tumor producing neutral mucins with pH > 2.5, which was the lower limit of detection for the Alcian Blue staining used.

### 3.4. Inhibition of Lactate Dehydrogenase Reverses the Growth Inhibition Caused by Lactate in EAC PDOs

**Figure 3:**
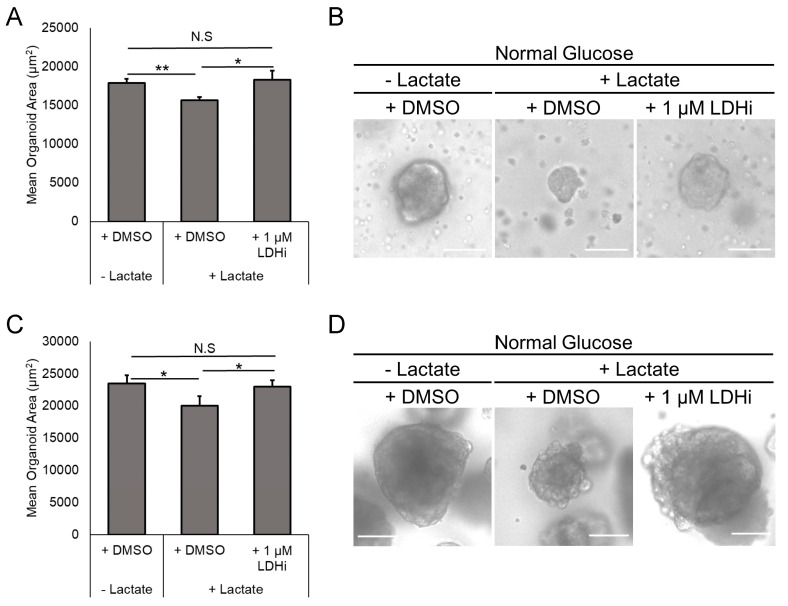
Inhibition of lactate dehydrogenase reverses lactate-driven growth inhibition. (**A**) Quantification of the size of EAC000 PDOs grown in the presence or absence of lactate and GSK2837808A, a lactate dehydrogenase inhibitor (LHDi), after 14 days. (**B**) Representative images of EAC000 PDOs grown in the labeled conditions. (**C**) Quantification of the size of HNEC001 PDOs grown in the presence or absence of lactate and GSK2837808A, a lactate dehydrogenase inhibitor (LHDi). (**D**) Representative images of HNEC001 PDOs grown in the labeled conditions. Error bars in (**A**,**C**) are standard deviations. * indicates *p* < 0.05 via Student’s *t*-test. ** indicates *p* < 0.01 via Student’s *t*-test. N.S indicates non-significant. Scale bars in (**B**,**D**) are 100 µm.

Lactate dehydrogenase (LDH) is a crucial enzyme in the metabolism of lactate and mediates a reversible reaction between the conversion of pyruvate to lactate. In humans, there are two predominant isoforms of LDH, LDHA, and LDHB. LDHA is thought to be the predominant form of LDH found in cancers and otherwise is primarily found in the liver and striated muscle [19]. It has a higher affinity for pyruvate and preferentially converts pyruvate to lactate [19]. LDHB is predominantly found in the heart and has a higher affinity for lactate [19]. We wondered if the growth inhibition seen in EAC PDOs was mediated by the action of LDH. We treated EAC PDOs grown in the presence of lactate with 1 µM GSK2837808A, an LDH inhibitor (LDHi). GSK2837808A is a selective LDHA inhibitor, but at higher concentrations (such as 1 µM) inhibits both LDHA and LDHB. Interestingly, treatment with GSK2837808A reversed the lactate-induced growth inhibition in both EAC000 and HNEC001 PDOs (Figure 3A–D), suggesting that LDH mediates the suppression of EAC cell proliferation in the lactate-exposed PDOs.

### 3.5. Treatment of EAC PDOs with Lactate Alters Tumor NADH/NAD+ Redox State

**Figure 4:**
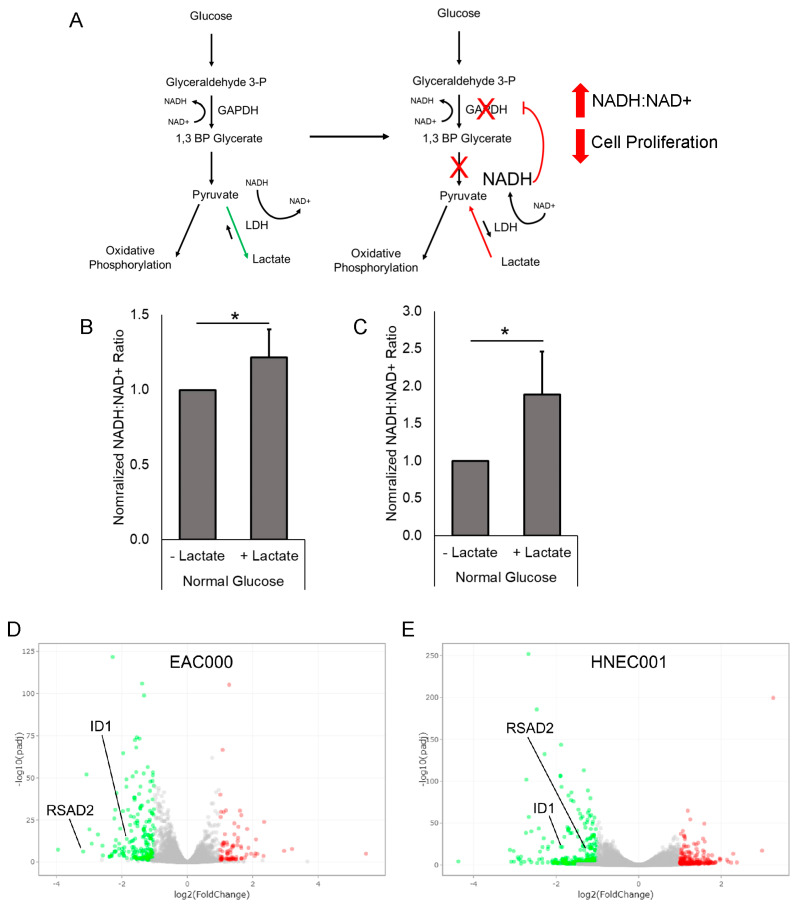
Lactate treatment of PDOs results in changes in glycolytic pathways. (**A**) Schematic of NADH/NAD+ alterations following lactate treatment. (**B**) Quantification of the NADH:NAD+ ratio in EAC000 PDOs grown in the listed conditions. (**C**) Quantification of the NADH:NAD+ ratio in HNEC001 PDOs grown in the listed conditions. (**D**) Volcano plot of differentially expressed genes in EAC000 PDOs treated with lactate compared to non-treated control. (**E**) Volcano plot of differentially expressed genes in HNEC001 PDOs treated with lactate compared to non-treated control. Significantly upregulated genes are shown as red dots, and downregulated genes are shown as green dots. Error bars in (**B**,**C**) are standard deviations. * indicates *p* < 0.05 via Mann–Whitney U test.

Glucose can be converted to pyruvate via glycolysis through multiple enzyme-mediated steps. In aerobic conditions, pyruvate can then be converted to Acetyl-CoA and then ultimately undergoes oxidative phosphorylation via the citric acid cycle. In anaerobic conditions, pyruvate can be converted to lactate via a reaction mediated by LDH, which also oxidizes Nicotinamide Adenine Dinucleotide + Hydrogen (NADH) to Nicotinamide Adenine Dinucleotide + (NAD +) in the process (Figure 4A). Notably, this reaction is reversible, with the reverse reaction converting lactate to pyruvate and the reduction of NAD+ to NADH (Figure 4A).

Given that the lactate-induced growth suppression we observed in EAC PDOs is reversed with inhibition of LDH, we wondered if the growth suppression was mediated by the reversal of the LDH-mediated reaction and conversion of lactate to pyruvate. To determine if lactate may be taken up the cells and converted to pyruvate, we employed a colorimetric assay to assess levels of NADH and NAD+ in the PDOs. Notably, we found that following treatment with lactate, there is an increase in the ratio of NADH to NAD+ (NADH:NAD+) compared to control PDOs (Figure 4B,C). This significantly increased NADH:NAD+ ratio was seen following lactate treatment in both EAC000 and HNEC001 PDOs (Figure 4B,C), indicative of increased concentrations of NADH relative to NAD+. This data indicates that higher concentrations of lactate result in a shift in tumor redox state, suggestive that the LDH-mediated conversion from lactate to pyruvate may be favored over the reverse reaction with a subsequent shift in the tumor NADH/NAD+ redox state with increased concentrations of NADH relative to NAD+ (Figure 4A).

### 3.6. RNAseq and Gene Ontology Analyses Reveal Lactate-Mediated Downregulation of ID1 and RSAD2 along with Enrichment for Metabolic and Immunological Pathways

We next performed bulk RNA sequencing of EAC000 and HNEC001 PDOs following lactate treatment to determine genetic and molecular mechanisms by which lactate may negatively regulate EAC cell proliferation. We specifically wondered if there are common differentially expressed genes that explain the physiological changes we observed. We identified 285 differentially expressed genes between lactate-treated and non-lactate-treated EAC000 PDOs and 521 differentially expressed genes between lactate-treated and non-lactate-treated HNEC001 PDOs (Supplemental Data S1). Notably, there are 28 common differentially expressed genes shared between EAC000 and HNEC001 PDOs (Supplemental Data S1). Among the shared genes are Inhibitor of Differentiation (ID1) and Radical S-Adenosyl Methionine Domain Containing 2 (RSAD2), both of which are significantly downregulated (Figure 4D). Both *ID1* and *RSAD2* have been implicated in regulating glycolytic pathways in cancer. *RSAD2* is a direct modulator of GAPDH activity, and *ID1* is known to upregulate multiple glycolysis enzymes (see Section 4). Notably, putative markers of EAC stem cells *LGR5* and *Musashi-1 (MSI1)* were not differentially expressed in EAC000 or HNEC001 following lactate exposure [20,21,22].

**Figure 5:**
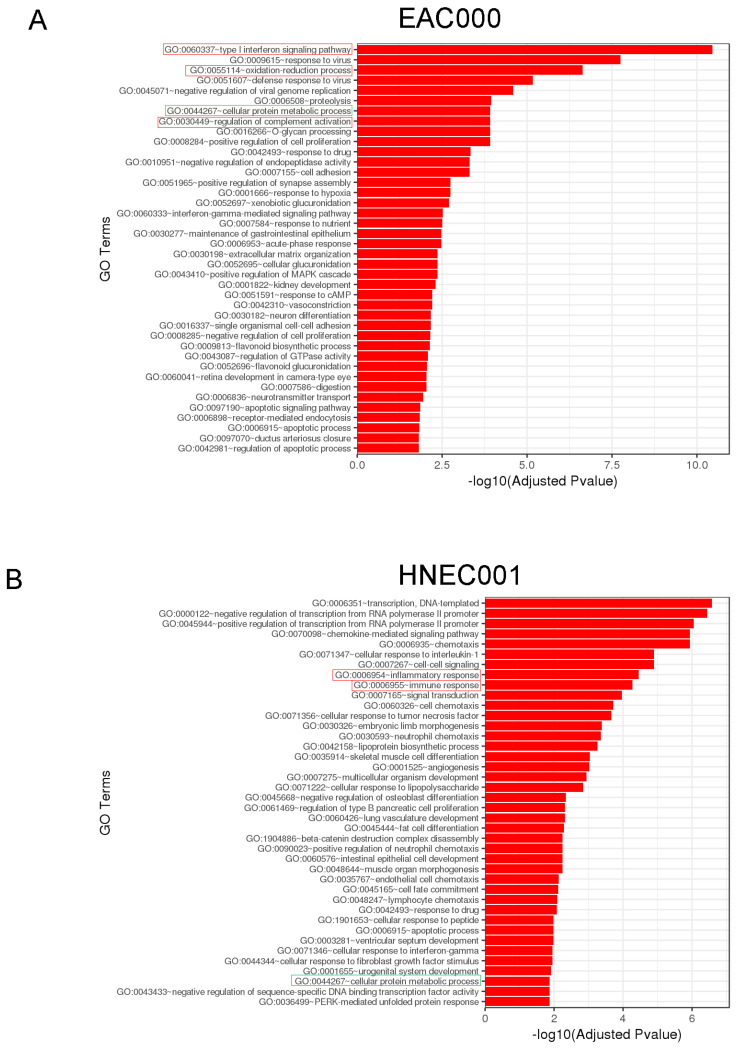
Gene ontology analysis reveals enrichment of genes associated with cellular metabolism and immune responses. (**A**) Gene ontology analysis for lactate-treated EAC000 PDOs compared to control. (**B**) Gene ontology analysis for lactate-treated HNEC001 PDOs compared to control. Pathways highlighted in green are shared between the two PDO lines. Immunological and inflammatory pathways are highlighted in red. Metabolic pathways are highlighted in green.

Further gene ontology pathway analysis of the RNA sequencing results showed lactate-mediated enrichment for multiple metabolic and immunological pathways. EAC000 PDOs showed enrichment for genes associated with the “type 1 interferon signaling pathway” and “regulation of complement activation” following lactate exposure (Figure 5A). Notably, lactate-treated EAC000 PDOs also had enrichment for genes associated with the “oxidation-reduction process” consistent with the shifts in oxidation/reduction status of NADH we observed above (Figure 5A). Lactate-treated HNEC001 PDOs had enrichment for genes associated with “inflammatory response” and “immune response” (Figure 5B). In both EAC000 and HNEC001 lactate-treated PDOs, there was an enrichment of genes associated with “cellular protein metabolic processes” indicative of metabolic shifts following lactate treatment (Figure 5A,B).

## 4. Discussion

Our results demonstrate that lactate can serve as a regulatory molecule in EAC growth and proliferation. Our data show that excess lactate suppresses cellular proliferation without significant changes in apoptosis. We demonstrated that this suppression of cellular proliferation is directly due to LDH as pharmacological suppression of LDH reversed the growth deficit in lactate-treated EAC PDOs.

Notably, our data suggest that the suppression of cellular proliferation may be through a shift in the oxidation/reduction balance of NAD+ and NADH. Specifically, we found that lactate treatment increases the reduction of NAD+ to NADH. Interestingly, lactate has also been shown to suppress the proliferation of T cells [23]. In T-cells, an overabundance of lactate results in a reversal of the LDH reaction, favoring the conversion of lactate to pyruvate and NAD+ to NADH (Figure 4A). The increased NADH is thought to disrupt the upstream glycolytic conversion of Glyceraldehyde 3-phosphate to 1,3 Bisphosphoglycerate (1,3 BP Glycerate), a reaction that is mediated by Glyceraldehyde-3-phosphate Dehydrogenase (GAPDH) and requires NAD+ (Figure 4A) [23].

Given we also observe an increased NADH:NAD+ ratio following lactate treatment in EAC PDOs following lactate treatment, a similar mechanism may explain the growth suppression we observe in EAC PDOs following lactate exposure. In support of this hypothesis, our RNA sequencing data identified *RSAD2* and *ID1*, regulators of GAPDH and glycolysis, both to be downregulated in EAC PDOs following lactate treatment.

*RSAD2* is a direct modulator of GAPDH activity and is typically activated by interferon in immune cells during viral infection [24,25]. The knockdown of *RSAD2* in macrophages results in increased activity of GAPDH, favoring the conversion of Glyceraldehyde 3-phosphate to 1,3 BP Glycerate [24,25]. Therefore, the downregulation of *RSAD2* we observe may represent a genetic response to overcome the inhibition of GAPDH caused by lactate via the shift in NAD+ to NADH. *ID1* has been observed to be overexpressed in liver tumors and is associated with the upregulation of multiple glycolysis enzymes, including Aldolase A, PFKB3, PFKL, PGAM1, and PDHK1 [26]. The observed downregulation of *ID1* in EAC PDOs following lactate treatment corresponds with a reduction in glycolysis and is consistent with the observed phenotype of decreased cellular proliferation. The exact mechanisms by which lactate regulates *RSAD2* and *ID1* remain unclear, and further investigation is warranted on whether and how lactate regulates glycolytic flux in EAC.

While NADH and NAD+ are well-established markers of cellular redox state, characterization of additional redox markers in lactate-treated PDOs, such as Glutathione and Glutathione Disulfide (GSH and GSSG) or Nicotinamide Adenine Dinucleotide Phosphate and Nicotinamide Adenine Dinucleotide Phosphate + Hydrogen (NADP and NADPH), would provide valuable insight into the global redox state of EAC PDOs following lactate treatment [27,28].

Interestingly, we find that excess lactate has variable effects in promoting morphological atypia in EAC PDOs, with some PDO lines gaining greater morphological atypia following lactate treatment and other lines unaffected by lactate. This effect is likely due to intrinsic genetic and mutational background differences between the EACs. This is supported by our RNA sequencing data, as there were only 28 common differentially expressed genes between EAC000 and HNEC001 EAC PDOs following lactate treatment and more than 700 differentially expressed genes that were unique to either EAC000 or HNEC001.

Our gene ontology analysis demonstrated the upregulation of inflammatory and immunomodulatory pathways in EAC PDOs following lactate exposure, suggesting a role for lactate in regulating these pathways. Supporting the potential interplay of lactate in regulating inflammation and immunomodulatory responses along with cellular proliferation is a recent study that demonstrated that co-culture of BE cell lines with lactate-producing bacterial species (including *Bifidobacterium* and *Lactobacillus* species) reduces the expression of key BE biomarkers such as p53 (a regulator of cellular proliferation), TNFα (a regulator of the NFκB inflammatory response), and IL-18 [29]. This study, along with our own, suggests that exogenous lactate is crucial in regulating a complex cellular proliferative and inflammatory tumor microenvironment that governs EAC and BE growth and dysplasia. Thus far, studies (including our own) are limited to identifying exogenous lactate as a potential inhibitor of BE and EAC growth and progression. Also, it remains unclear whether intrinsic lactate (produced by the tumor) has a similar effect. In other cancer types, intrinsic lactate has been proposed to serve as an energy source for the proliferation of cancer cells by serving as a crucial carbon source for the TCA cycle [30]. Additionally, intrinsic lactate has been demonstrated to serve as a carbon source for lipid synthesis in other cancer types [31]. Notably, intrinsic lactate produced by cancer cells through the Warburg effect has been shown to enrich the tumor microenvironment and suppress immune responses [12,32]. Future studies utilizing EAC PDO co-cultures with immune cells and fibroblasts are warranted to investigate the interplay between lactate, immune cells, and the tumor microenvironment.

## 5. Conclusions

Prior studies have predicted an increase in lactate during the dysplastic progression of a healthy esophagus from BE to EAC [8]. The exact role of lactate in the pathogenesis of BE and EAC has not yet been well characterized. Our study shows that exogenous lactate can serve as a regulatory molecule in EAC growth and proliferation. Excess exogenous lactate suppresses EAC growth accompanied by a shift in the NADH/NAD+ redox state of the tumor, which may, in turn, inhibit glycolysis by the mechanisms described. These alterations in metabolic pathways may be mediated by *ID1* and *RSAD2* in addition to potential alterations in immunological and inflammation pathways. Overall, our study suggests that exogenous lactate from the tumor microbiome may serve to inhibit the progression of EAC. Further investigation into the mechanisms by which lactate regulates glycolysis is warranted, specifically the role of *RSAD2* and *ID1*. Additional bacterial metabolites, such as Butyrate and L-tryptophan, have also been predicted to be altered when comparing a healthy esophagus to BE and EAC [8]. Further studies into these additional microbiome-derived metabolites and their role in BE and EAC carcinogenesis are also warranted. Through these further studies, esophageal microbiome and metabolite profiling may serve as a promising tool for risk stratifying BE and EAC progression.

## Data Availability

The original contributions presented in the study are included in the article/supplementary material, further inquiries can be directed to the corresponding author.

## Supplementary Materials

The following supporting information can be downloaded at: https://www.mdpi.com/article/10.3390/biom14091195/s1, Supplemental Figure S1. Lactate treatment suppresses growth of EAC PDOs after 10 days of treatment. Supplemental Figure S2. Delay in lactate exposure still results in smaller EAC000 PDOs. Supplemental Figure S3. Addition of Lactate does not significantly alter pH of media. Supplemental Figure S4. Lactate treatment increases neoplastic changes in HNEC001 but not EAC000. Supplemental Figure S5. Expression of metaplastic markers in EAC PDOs. Supplemental Figure S6. Clinical and pathological characterization of tumor samples from which PDOs were derived. Supplemental Data S1: Excel file identifying differentially expressed genes in EAC000 and HNEC001 following lactate treatment through RNA sequencing.
