# Supplementary material for: Lactate Suppresses Growth of Esophageal Adenocarcinoma Patient-Derived Organoids through Alterations in Tumor NADH/NAD+ Redox State
Source: Biomolecules. 2024 Sep 22;14(9):1195. doi: 10.3390/biom14091195 (PMC11430592; doi:10.3390/biom14091195)
Supplement: Supplementary file 1 [file biomolecules-14-01195-s001.zip › Supplemental Material Legends.pdf]

Supplemental Figure S1. Lactate treatment suppresses growth of EAC PDOs after 10 days of treatment. A. Schematic indicating experimental procedure. B. Quantification of mean organoid area of EAC000 PDOs after 10 days of lactate treatment. C. Quantification of mean organoid area of HNEC001 PDOs after 10 days of lactate treatment. *indicates $p<0.05$ via Student's t-test. ** indicates $p<0.01$ via Student's t-test. Error bars in B and C are standard deviation.

Supplemental Figure S2. Delay in lactate exposure still results in smaller EAC000 PDOs. A. Schematic showing delayed treatment of lactate to EAC000 PDOs. B-C. Quantification of OFR (B) and mean organoid area (C) following delayed treatment of lactate. D. Representative images of EAC000 PDOs at day 14 following delayed exposure to lactate. Error bars in (B) and (C) are standard deviation. **$p<0.01$. N.S denotes non-significant. Scale bars in (D) are 100 µm.

Supplemental Figure S3. Addition of Lactate does not significantly alter pH of media. The pH of media was measured immediately prior to addition to PDO culture and after 3 days of culture with PDOs.

Supplemental Figure S4. Lactate treatment increases neoplastic changes in HNEC001 but not EAC000. EAC000 and HNEC001 EAC PDOs were grown for 14 days. A. Hematoxylin and eosin staining of EAC000 PDOs under various conditions. B. Quantification of neoplasia scores in EAC000 under the indicated conditions. C. Hematoxylin and eosin staining of HNEC001 PDOs under various conditions. D. Quantification of neoplasia scores in HNEC001 PDOs under the indicated conditions. E. Histopathologic scoring system used to score neoplasia in the PDOs. N.S indicates non-significant. ** indicates $U<0.01$ by Mann-Whitney U test. *** indicates $U<0.001$ by Mann-Whitney U test. N=20 PDOs examined for each condition.

Supplemental Figure S5. Expression of intestinal metaplastic markers in EAC PDOs. A. Alcian blue expression and immunofluorescent expression of CDX2 and Cytokeratin 8 (K8) in EAC000 PDOs grown under the indicated treatment groups. B Quantification of CDX2 expression in EAC000 PDOs under various growth conditions. C. Quantification of K8 expression in EAC000 PDOs under various growth conditions. D. Alcian blue expression and immunofluorescent expression of CDX2 and Cytokeratin 8 (K8) in HNEC001 PDOs grown under the indicated treatment groups. E. Quantification of CDX2 expression in HNEC001 PDOs under various growth conditions. F. Quantification of K8 expression in HNEC001 PDOs under various growth conditions. G. Alcian blue expression and immunofluorescent expression of CDX2 and Cytokeratin 8 (K8) in EAC011 PDOs grown under the indicated treatment groups. H. Quantification of CDX2 expression in EAC011 PDOs under various growth conditions. I. Quantification of K8 expression in EAC011 PDOs under various growth conditions. Scale bars in A, D, and G are 100 µm. Error bars in B, C, E, F, H, and I are standard deviation. N.S indicates non-significant by student's t-test.

Supplemental Figure S6. Clinical and pathological characterization of tumor samples from which PDOs were derived.

Supplemental Data S1. Excel file identifying differentially expressed genes in EAC000 and HNEC001 following lactate treatment through RNA sequencing.
